# The Role of ^18^F-FDG PET/CT in Evaluating the Efficacy of Radiofrequency Ablation in Metastatic and Primary Liver Tumors: Preliminary Results

**DOI:** 10.4274/mirt.galenos.2020.63634

**Published:** 2021-02-09

**Authors:** Gabriela Mateva, Stoyan Handzhiev, Irena Kostadinova

**Affiliations:** 1 Acibadem City Clinic Mladost, Clinic of Nuclear Medicine, Sofia, Bulgaria; 2 Acibadem City Clinic Mladost, Clinic of Gastroenterology, Sofia, Bulgaria

**Keywords:** Radiofrequency ablation, PET/CT, CEUS, metastatic liver lesions, primary liver tumors

## Abstract

**Objectives:**

The aim of the study was to investigate the role of ^18^fluorine-fluorodeoxyglucose (^18^F-FDG) positron emission tomography/computed tomography (PET/CT) for evaluating the efficacy of radiofrequency ablation (RFA) in primary and metastatic liver tumors compared with contrast-enhanced ultrasound examination (CEUS) and to find its place in overall staging and the follow-up diagnostic algorithm.

**Methods:**

PET/CT examinations were performed 2 months after RFA for 20 patients with a total of 34 liver lesions. CEUS was performed within 10 days after PET/CT, and the results were compared. Seven patients were staged with PET/CT and the others with a contrast-enhanced CT.

**Results:**

A total of 48 ^18^F-FDG PET/CT examinations were performed. We observed complete response in 8 patients (40%), 2 patients (10%) had stable disease, one (5%) had partial response, and 9 patients (45%) had progression (including 2 cases with extrahepatic involvement). Compared with CEUS, there was a mismatch in 3 cases. Five patients underwent additional RFA for 7 lesions.

**Conclusion:**

According to our preliminary data, PET/CT may be a valuable method, with comparable or eventually even better sensitivity than CEUS, for early evaluation of the efficacy of RFA for the treatment of metastatic and primary liver lesions and planning of future treatment. PET/CT might be recommended as a staging method before undergoing RFA of liver lesions for determining the local extent of the disease in the liver in combination with CEUS with an advantage in visualization of extrahepatic involvement. However, more patients need to be investigated in order to demonstrate and confirm the obtained results with certainty.

## Abstract (tr)

**Amaç:**

Bu çalışmanın amacı, primer ve metastatik karaciğer tümörlerinde radyofrekans ablasyonunun (RFA) etkinliğini değerlendirmede ^18^flor-florodeoksiglukoz (^18^F-FDG) pozitron emisyon tomografisi/bilgisayarlı tomografinin (PET/BT) rolünü kontrastlı ultrason tetkikine (CEUS) kıyasla araştırmak ve genel evreleme ve takip tanı algoritmasındaki yerini bulmaktır.

**Yöntem:**

Toplam 34 karaciğer lezyonu olan 20 hastaya RFA’dan 2 ay sonra PET/BT tetkikleri yapıldı. PET/BT’den sonraki 10 gün içinde CEUS yapıldı ve sonuçlar karşılaştırıldı. Yedi hasta PET/BT ile diğerleri ise kontrastlı BT ile evrelendirildi.

**Bulgular:**

Toplam 48 adet ^18^F-FDG PET/BT incelemesi yapıldı. Sekiz hastada (%40) tam yanıt, 2 hastada (%10) stabil hastalık, 1 hastada (%5) kısmi yanıt ve 9 hastada (%45) progresyon (ekstrahepatik tutulumlu 2 olgu dahil) gözlemlendi. CEUS ile karşılaştırıldığında, 3 olguda uyumsuzluk bulundu. Beş hastaya, toplam 7 lezyon için ek RFA uygulandı.

**Sonuç:**

Ön verilerimize göre PET/BT, metastatik ve primer karaciğer lezyonlarının tedavisinde RFA’nın etkinliğinin erken değerlendirilmesi ve gelecekteki tedavinin planlanması için CEUS ile karşılaştırılabilir veya sonuç olarak daha iyi hassasiyete sahip değerli bir yöntem olabilir. PET/BT, ekstrahepatik tutulumun görselleştirilmesinde avantajlı olması ile birlikte, CEUS ile birlikte karaciğerdeki hastalığın lokal boyutunu belirlemede karaciğer lezyonları için RFA öncesinde bir evreleme yöntemi olarak önerilebilir. Bununla birlikte, elde edilen sonuçları kesin olarak göstermek ve doğrulamak için daha fazla hastanın araştırılması gerekir.

## Introduction

Radiofrequency ablation (RFA) of liver lesions is a minimally invasive treatment option for patients with primary and metastatic hepatic tumors. RFA of liver lesions is performed under visual control, most often using ultrasound-rarely computed tomography (CT)-and there is also a report of the procedure being performed under positron emission tomography (PET)/CT control (1).

The main indications for thermal ablation include unresectable liver lesions; combination with hepatectomy as an additional treatment; patients with significant medical comorbidities or poor performance status; and small (<3 cm), solitary lesions, which would otherwise necessitate a major liver resection (2).

PET/CT is not a standard method for patient follow-up after RFA, but a literature review and our experience suggest that it is applicable, highly sensitive and specific, and might even have advantages over other imaging methods (contrast-enhanced ultrasound and CT) in certain clinical situations.

The aim of the study is to investigate the role of ^18^fluorine-fluorodeoxyglucose (^18^F-FDG) PET/CT for evaluating the efficacy of RFA in primary and metastatic liver tumors and to compare the results with those of contrast-enhanced ultrasound examination (CEUS) in overall staging and the follow-up diagnostic algorithm.

## Materials and Methods

Our initial experience was an examination of 20 patients, 9 men and 11 women, with a mean age of 63 years, during the period 2017-2019, with a total of 34 liver lesions, with the following primary tumors: Six with colon cancer, 5 with breast cancer, 3 with pancreatic cancer, 2 with hepatocellular carcinoma (HCC), 2 with stomach cancer, one cancer of the ampulla of Vater, and 1 with carcinoma of the epipharynx. PET/CT examinations were performed 2 months after RFA. We used a standard scanning protocol: Intravenous injection of ^18^F-FDG 2-2.2 MBq per kilogram and whole-body scanning (calvaria to mid-thigh), after 60 minutes of rest, 2 minutes per bed position combined with dynamic CT scanning with a 2.5 mm slice thickness. For interpretation of the results and evaluation of efficacy of RFA, we used qualitative/visual and quantitative criteria: Maximum standard uptake value (SUV_max_) values and changes in lesion size (3).

The RFA generator was used to apply a power of 40 kW for 5,10, 15, or 20 min, depending on the size of the lesion, with 1 and 2 working antennas with a working electrode length of 15-20-25 mm in monopolar or bipolar mode. Ultrasound navigation was performed with contrast-enhancing mode software, with a mechanical index of -0.18, and bolus application of 2.5 mL contrast agent. The lesion size was measured, and the contrast enhancement was observed.

In all cases, a CEUS was performed within 10 days before or after PET/CT, and the results were compared.

For 7 of the patients, a staging PET/CT was performed, and the others already had a recent contrast-enhanced CT, which was used for staging.

The exclusion criteria were extrahepatic involvement and cases where the number, size, or location of the hepatic lesions precluded total ablation.

The study protocol was approved by the Ethics Committee of Acibadem City Clinic (date: 12.10.2018, approval no: 11-07-80). Before undergoing the studies and RFA, all patients signed informed consent forms, agreeing that the obtained data be used for scientific purposes.

Statistical analysis was not performed since the number of included subjects was not suitable for drawing reliable conclusions.

## Results

A total of 48 ^18^F-FDG PET/CT examinations were performed. The patients were restaged for local status after RFA of liver lesions and for distant metastases with 41 follow-up PET/CT studies.

We observed complete response in 8 patients (40%), 2 patients (10%) had stable disease, 1 (5%) had partial response, and 9 patients (45%) had progression (including 2 cases with extrahepatic involvement).

Compared to CEUS, there was a mismatch in three cases. In 2 patients where PET/CT showed increased metabolic activity of lesions, respectively, interpreted as progression, in CEUS the same lesions were considered as necrosis and good therapeutic effect. In 1 case, PET/CT showed 2 metabolically active lesions, while CEUS could identify only one pathological liver lesion.

As a result of the follow-up, including PET/CT, five patients underwent additional RFA for 7 lesions.

In 5 patients that were clinically intended for treatment with RFA, the ^18^F-FDG PET/CT staging revealed additional extrahepatic lesions; therefore, the treatment plan was changed to a systematic instead of local approach, and the procedure was abandoned. These patients were excluded from the observation group.

## Discussion

**Figure 1:**
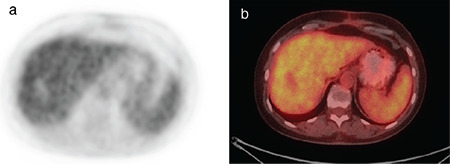
^18^F-FDG-PET/CT of a patient with complete response to the treatment of a liver metastasis of breast cancer 2 months post RFA. The photophenic zone in the 4b liver segment on the metabolic images corresponds with coagulation necrosis. a) PET post-treatment image, axial slice on the level of the 4b segment of the liver, b) fused axial slice on the level of the 4b segment of the liver, post-treatment image ^18^F-FDG: ^18^Fluorine-fluorodeoxyglucose, PET/CT: Positron emission tomography/computed tomography, RFA: Radiofrequency ablation

The use of RFA for liver metastases has been reported to be effective and safe by many authors (4). It does not have as good results in terms of disease-free and long-term survival as surgical treatment (5), but for patients who are not eligible for surgery, it is a valuable non-invasive treatment option. Studies on the long-term survival of non-surgically treated patients with hepatic colorectal metastases who underwent RFA reported a 1-year survival rate of 86%-99%, a 3-year survival rate of 46%-68%, and a 5-year survival rate of 24%-44% (4). The expected effect of RFA is based on thermal destruction of the tumor by variable radiofrequency waves, delivered locally, most often percutaneously, by a special electrode, heating the tissues/aiming to reach 60 °C, which leads to coagulation necrosis (Figure 1).

**Figure 2:**
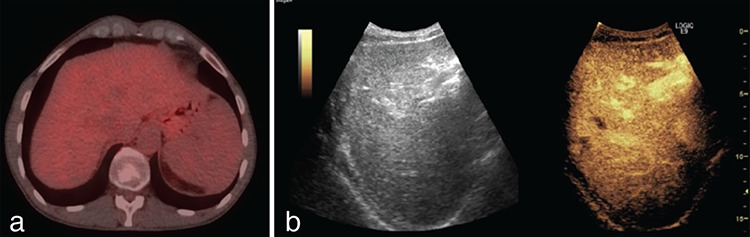
A patient with liver metastasis from colorectal cancer with complete response 2 months after RFA. The PET/CT images show no metabolic or morphological abnormalities in the area of the ablated lesion. CEUS shows full necrosis of the lesion in the 7^th^ liver segment. a) Axial fused post-treatment image on the level of 7^th^ liver segment, b) CEUS post-treatment image RFA: Radiofrequency ablation, PET/CT: Positron emission tomography/computed tomography, CEUS: Contrast-enhanced ultrasound examination

In the patient group we investigated, RFA ablation was performed under direct ultrasound control with contrast enhancement, and the established clinical protocol for follow up and the evaluation of treatment response was also with CEUS. Contrast enhancement significantly increases the certainty and efficiency of the method. According to the guidelines of the European and World Association for the Application of Ultrasound Methods in Biology and Medicine from 2012, CEUS is indicated for monitoring the effect of RFA, performed on liver tumors, based on comparable results with CT and magnetic resonance imaging (MRI) (6), with sensitivity and specificity reaching 80%-90%, according to the literature data (7). It is inexpensive, accessible, not connected with any additional radiation burden for the patient, and it is possible to perform follow-up studies in a very short period before or after PET/CT and, if needed, serial additional studies. The final result is greater certainty for the therapeutic effect of RFA (Figure 2).

**Figure 3:**
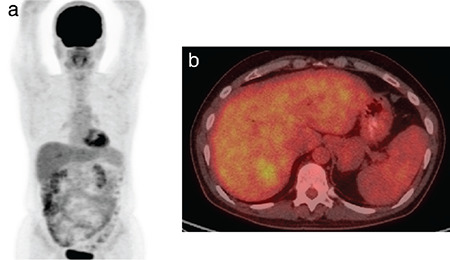
A baseline PET/CT examination of a patient with a solitary liver metastasis from pancreatic carcinoma in the 7^th^ liver segment with SUV_max_ 3.6, which was treated with RFA a week after this study. a) MIP projection, pre-treatment image, b) axial fused pre-treatment image on the level of the 7^th^ liver segment PET/CT: Positron emission tomography/computed tomography, RFA: Radiofrequency ablation, SUV_max_: Maximum standard uptake value, MIP: Maximum intensity projection

**Figure 4:**
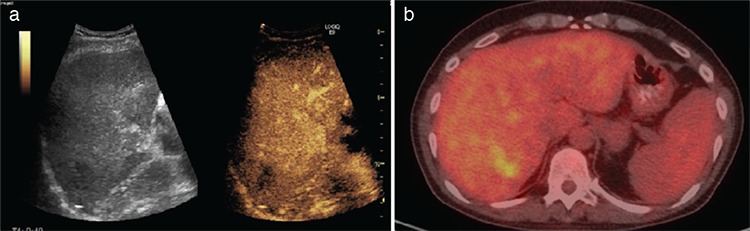
Results from the restaging PET/CT and CEUS of the same patient performed 2 months after the RFA were discrepant. The CEUS showed complete necrosis, interpreted as complete response, while on PET/CT there was a persistent metabolically active lesion with partial reduction of the size and activity (SUV_max_ 3), interpreted as partial response. a) CEUS, post-treatment image showing complete necrosis, b) fused axial image on the level of the 7^th^ liver segment, post-treatment, showing a persistent metabolically active lesion with partial reduction of the size and activity PET/CT: Positron emission tomography/computed tomography, CEUS: Contrast-enhanced ultrasound examination, RFA: Radiofrequency ablation, SUV_max_: Maximum standard uptake value

To get the most out of the obtained PET/CT information and to perform a reliable interpretation, it is important to select the patients correctly. Most often, patients with tumors that are not expected to utilize ^18^F-FDG (such as differentiated HCC, mucinous colon carcinomas, clear cell renal cell carcinoma, etc.) should not be examined. All patients undergoing RFA of liver lesions, which are intended to be followed up with PET/CT, should have a baseline study before the procedure so that the metabolic changes in the lesion in addition to the morphological data can be tracked, and a more precise and complex treatment response decision can be made (Figure 3, 4).

As stated earlier, five patients were ruled out of the treated and investigated group (approximately 40% of the patients referred for staging) because the PET/CT showed a greater extent of the disease such as additional metastases in lymph nodes, lung, and bones or more extensive liver involvement than initially expected, and the management plan was changed.

To avoid false-positive results, e.g., inflamed tissue around the region of ablation, the timing of the PET/CT examination after the ablation is very important; it should either be before the beginning of the reparative processes or after they have resolved. The protocol we have adopted is to scan patients around 2 months after treatment. However, according to some data, PET/CT could be a very useful methodology for evaluating ablation immediately after the procedure-up to 48 hours, with high (up to 100%) (8) sensitivity for a residual tumor, much better than all other imaging methods. This is due to the loss of the ability of the ablation-treated cells to accumulate glucose and they are imaged as a photopenic zone. The concomitant peripheral hyperemia, in all other imaging studies, visible as peripheral enhancement, is very difficult to distinguish from a residual tumor. The nuclear medicine examination does not show increased glucose metabolism in such a short period; accordingly, the presence of an area with increased metabolic activity up to the 48^th^ hour on ^18^F-FDG PET/CT is most likely due to a residual tumor and could be further re-ablated (9,10).

According to other authors, PET/CT examinations must be performed 2 months after RFA (9,11), as this is the shortest period that allows for evaluation of the treatment effect with a low likelihood of false-positive lesions due to active necrotic and inflammatory changes after treatment. We have accepted this protocol as it is more convenient than other approaches and safer for patients in terms of the radiation burden.

A study by Chen et al. (12) suggested that ^18^F-FDG is superior to MRI and/or CT, with overall accuracies of 87.9%, 75.0%, and 64.3%, respectively, and is more cost-effective in post-RFA hepatic tumor assessment. The average scan numbers for PET, MRI, and CT to achieve a final accurate diagnosis were 1.121, 1.316, and 1.250, respectively. As stated earlier, CEUS is reported to have similar sensitivity and specificity of CT and MRT for assessment of liver metastases (7), thus it could be expected that PET/CT would also be superior to CEUS. However, there are no reliable data comparing directly the performance of both methods for this indication.

**Figure 5:**
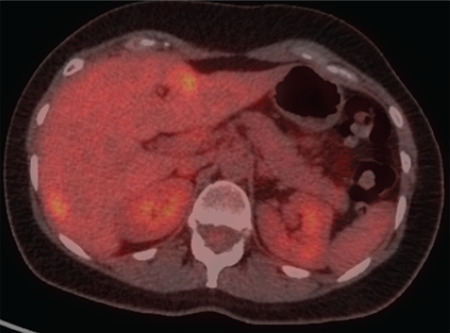
A baseline PET/CT of a patient with 2 liver metastases from breast cancer, who was treated with RFA. Pre-treatment axial fused slice on the level of liver segments 2 and 7, showing metabolically active liver lesions respectively with SUV_max_ 5.2 and SUV_max_ 4.3 PET/CT: Positron emission tomography/computed tomography, RFA: Radiofrequency ablation, SUV_max_: Maximum standard uptake value

**Figure 6:**
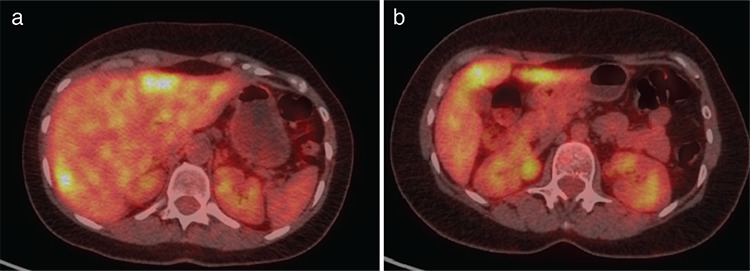
A restaging PET/CT of the same patient, showing progression 2 months after RFA with new liver lesion and increased size and activity of the ablated lesions. a) Fused axial post treatment image on the level of liver segments 2 and 7, showing metabolically active liver lesions respectively with SUV_max_ 5.5 and SUV_max_ 5.8. b) Fused axial post treatment image on the level of liver segment 4b, showing a new metastatic lesion with SUV_max_ 4.7 PET/CT: Positron emission tomography/computed tomography, RFA: Radiofrequency ablation, SUV_max_: Maximum standard uptake value

It is expected that in many patients, the disease will progress over time, despite treatment, so long-term follow-up is required and should include not only local treatment evaluation of the liver with US but also whole-body PET/CT scans, which have the potential to give more accurate information on the disease (Figure 5, 6).

According to our experience, patient monitoring and follow-up strategies need to be carried out by a multidisciplinary team consisting of a gastroenterologist, nuclear medicine specialist, oncologist, surgeon, and radiologist in order to determine further management. It is important that RFA, although intended to be a substitute, does not exclude the possibility of any subsequent surgical treatment if it is clinically appropriate.

If needed, RFA must be combined with systemic therapy (chemotherapy, hormones, immuno-, targeting, etc.), with an expected improvement in survival. There are active studies that specifically investigate certain therapies in combination with RFA of liver lesions, such as EORTC-1560-GITCG, which evaluates the effects of immunotherapy with durvalumab and tremelimumab in combination with RFA or stereotactic radiosurgery. However, all of them are at an early stage, and further results are expected.

A few studies are available (13) on the role of SUV values as a prognostic biomarker prior to the ablation of liver lesions. At this stage, it has been found that low baseline SUV values for colorectal cancer correlate with prolonged liver failure-free survival.

### Study Limitations

The main limitation of the study is the low number of included patients, which is not sufficient to make general conclusions and recommendations. However, the literature review showed that this is a common limitation of all of the research performed on this topic, so any additional data could make a contribution.

The lack of baseline PET/CT in many of the cases was challenging in terms of treatment response evaluation. However, in clinical settings, such as the one in which this study was conducted, there are some practical limitations (cost, time effectiveness, radiation burden, etc.), and having a PET/CT performed before each RFA was not achievable. When possible, a PET/CT should be conducted within a month before the ablation to select the patients that are likely to benefit from the treatment and to have a basis for comparison with the following PET/CT studies.

The possibility of false-negative results must also be considered for reliable and objective evaluation, most often in small (less than 5-10 mm). A more convincing result can be achieved by late scanning or additional software image processing. In both cases the aim is to improve the ratio of suspected lesions to background activity. The most common false-positive results that we should keep in mind are active reparative changes and liver abscesses (14).

## Conclusion

According to our preliminary data, PET/CT may be a valuable method, with comparable or eventually even better sensitivity than CEUS, for early evaluation of the efficacy of RFA for the treatment of metastatic and primary liver lesions and planning of future treatment.

PET/CT might be recommended as a staging method before undergoing RFA of liver lesions to determine the local extent of the disease in the liver in combination with CEUS with an advantage in visualization of extrahepatic involvement.

However, more patients must investigated in order to demonstrate and confirm the obtained results with certainty.
